# Progressive multifocal leukoencephalopathy as the initial presentation of leukemia in a chemotherapy-naive patient

**DOI:** 10.1055/s-0046-1822635

**Published:** 2026-05-12

**Authors:** Patricia Orozco-Puga, Naomi Nazareth Becerra-Aguiar, César Ibarra-de la Torre, Amado Jiménez-Ruiz, José Luis Ruiz-Sandoval

**Affiliations:** 1 Universidad de Guadalajara, Centro Universitario de Ciencias de la Salud, Guadalajara, Mexico.; 2 Hospital Civil de Guadalajara, Unidad Hospitalaria Fray Antonio Alcalde, Stroke & Cerebrovascular Disease Clinic, Guadalajara, Mexico.

**Figure 1:**
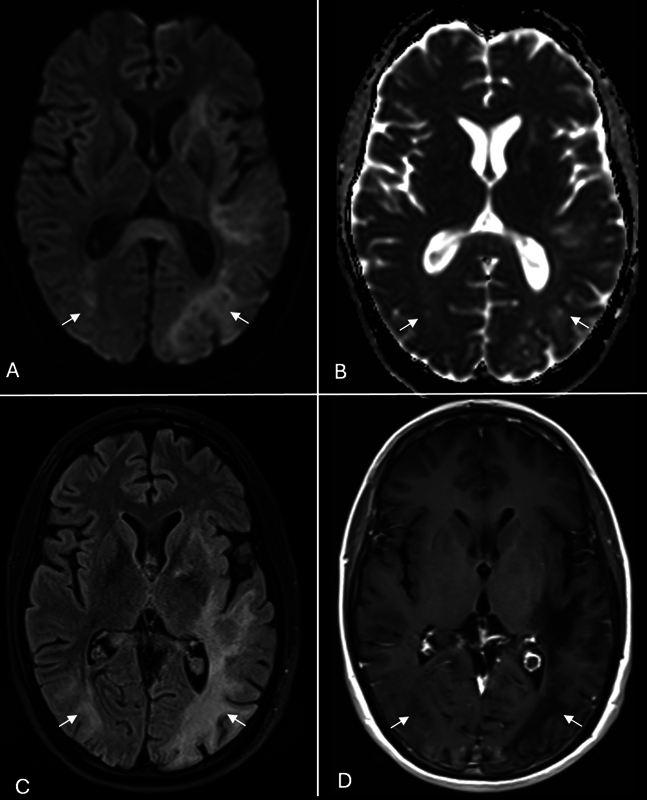
Brain magnetic resonance imaging (MRI). ( **A** ) Diffusion-weighted imaging (DWI) showing hyperintense lesions (White arrows) with a peripheral rim of diffusion restriction, ( **B** ) with corresponding low signal on the apparent diffusion coefficient (ADC) map. ( **C** ) Fluid-attenuated inversion recovery (FLAIR) images demonstrating multifocal, asymmetric hyperintense signals in the subcortical and deep white matter (white arrows), predominantly in the parieto-occipital regions with ill-defined margins and no appreciable mass effect. ( **D** ) Postcontrast T1-weighted imaging revealing no contrast enhancement (white arrows).

A previously healthy 46-year-old man presented with subacute cognitive changes and progressive visual decline, weight loss and behavioral changes. Neurological examination showed isolated language impairment. Brain magnetic resonance imaging (MRI) revealed diffuse multifocal white matter hyperintensities ( Figure 1 ). Laboratory testing demonstrated marked lymphocytosis, and flow cytometry confirmed B-cell chronic lymphocytic leukemia. Lumbar puncture showed a positive John Cunningham virus (JC) result to polymerase chain reaction (PCR), confirming a diagnosis of progressive multifocal leukoencephalopathy (PML) preceding chronic lymphocytic leukemia (CLL) diagnosis.

It is rare for PML to be the first manifestation of occult hematologic malignancy 1 2 due to hypogammaglobulinemia and T-cell dysfunction. Indolent CLL may cause immune dysregulation before symptoms. Unexplained demyelinating lesions warrant evaluation for immunodeficiency, even in chemotherapy-naive patients. 3 4
